# Metabolically Healthy Obesity Is a Misnomer: Components of the Metabolic Syndrome Linearly Increase with BMI as a Function of Age and Gender

**DOI:** 10.3390/biology12050719

**Published:** 2023-05-15

**Authors:** Yonit Marcus, Elad Segev, Gabi Shefer, David Eilam, Galina Shenkerman, Assaf Buch, Shani Shenhar-Tsarfaty, David Zeltser, Itzhak Shapira, Shlomo Berliner, Ori Rogowski

**Affiliations:** 1 The Sagol Center for Epigenetics of Metabolism and Aging, Sourasky Medical Center, Tel Aviv 6423906, Israel; yonit.marcus@gmail.com (Y.M.); elad1segev@gmail.com (E.S.);; 2 Institute of Endocrinology, Metabolism and Hypertension, Tel-Aviv-Sourasky Medical Center, Tel Aviv 6423906, Israel; 3 Sackler Faculty of Medicine, Tel Aviv University, Tel Aviv 6997801, Israel; 4 Department of Applied Mathematics, Faculty of Science, Holon Institute of Technology, Holon 5810201, Israel; 5 School of Zoology, George S. Wise Faculty of Life-Sciences, Tel-Aviv University, Tel Aviv 6997801, Israel; 6 Departments of Medicine and Preventive Medicine, Tel Aviv-Sourasky Medical Center, Tel Aviv 6423906, Israel

**Keywords:** metabolic syndrome, obesity, hypertension, metabolically healthy obesity, normometabolic obesity

## Abstract

**Simple Summary:**

Driven by observations that some obese, even morbidly obese subjects show none of these features, evidence has been presented to support the existence of “normometabolic obesity” (NMO). The concept underlying this “syndrome” is that certain obese subjects are somehow protected from the MS sequels of obesity. Our new findings presented here are: (A) “Metabolically-healthy-obesity” is rare in subjects with high BMI and declines with age; (B) Hypertension is the most common MS component in men with MS1-4; while in women, MS components are seen at an older age than men for the same BMI. Metabolic health declines with age and BMI in nearly all obese subjects. This cross-sectional study predicts that in men early rise in blood pressure and actual hypertension precedes the subsequent emergence of additional components of the MS. The continuous weight-related change in MS components argues against dichotomization of obesity into normal/abnormal in terms of its metabolic and vascular sequels. Further, because the harms of obesity may be delayed but significant, a permissive approach towards the metabolically healthy state appears ill-advised.

**Abstract:**

Objectives: We aimed to examine the relationships between body mass index (BMI) and metabolic syndrome (MS) components as a function of age and gender across weight categories. Methods: This cross-sectional study included 19,328 subjects who participated in a health-screening program. We analyzed 14,093 apparently healthy subjects with a BMI ≥ 18.5 kg/m^2^ (ranging from 18.5 to 46 kg/m^2^). Results: At a BMI of 18.5 kg/m^2^, 16% of subjects had one or more MS components (MS ≥ 1). The number of MS components increased linearly with BMI. The most prevalent components for MS1-4 were hypertension (in men) and increased waist circumference (in women). Among 6391 non-obese subjects with MS = 0, there was a linear increase in blood pressure, glucose, and triglycerides, as well as a decline in high-density lipoprotein cholesterol, as BMI increased. In 2087 subjects with a BMI ≥ 30 kg/m^2^, a true normometabolic state (MS = 0) was observed in only 7.5%, declining to less than 1% at a BMI ≥ 36 kg/m^2^ (ATP criteria). Women were metabolically protected relative to men between the ages of 30 and 50 years. Conclusions: (A) MS components increase linearly with BMI from the lowest normal BMI and continue to increase with age and BMI; (B) metabolically healthy obesity is rare in subjects with a high BMI and declines with age; (C) hypertension is the most common component in men; and (D) in women, MS components are seen at older ages than in men for the same BMI. Metabolic health declines with age and BMI in nearly all subjects with obesity.

## 1. Introduction

The increasing worldwide prevalence of obesity is generally perceived as the key factor in the alarmingly rising incidence of type 2 diabetes [1,2]. The transition from obesity to deranged glucose control, diabetes, and cardiovascular disease (CVD) is gradual and is perhaps best addressed by the term “the metabolic syndrome” (MS), which reflects the association of obesity, particularly central obesity, with several of its complications, including hypertension. Although there is some controversy regarding the association between MS and CVD, there is a wealth of data pointing to metabolic syndrome as a precursor of cardiovascular disease. It is even suggested that “identification of individuals with the metabolic syndrome may provide opportunities to intervene earlier in the development of shared disease pathways that predispose individuals to both CVD and diabetes” [3,4,5].

Reflective of the tendency of metabolic and cardiovascular effects of central obesity to cluster, the metabolic syndrome, MS, has been generally defined by the presence of at least three of the following risk determinants: (1) increased waist circumference (WC) as defined by the US Adult Treatment Panel (ATPIII; >102 cm for men; >88 cm for women) or by the International Diabetes Federation (IDF) for Europid subjects (men > 94 cm, women > 80 cm); (2) triglycerides ≥ 1.65 mmol/L; (3) low HDL cholesterol (men < 1.03 mmol/L, women < 1.29 mmol/L); (4) hypertension (≥130/≥85 mmHg); and (5) impaired fasting glucose (≥6.1 mmol/L 5.55 mmol/L ATPIII) or diabetes [6]. Of note is that among the components of metabolic syndrome, hypertension and impaired fasting glucose were shown to be significantly associated with all-cause and cardiovascular mortality [7].

Driven by observations that some obese, even morbidly obese, subjects show none of these features, evidence has been presented to support the existence of “normometabolic obesity” (NMO). Metabolically healthy obesity, or NMO, is generally defined as obesity without metabolic syndrome (MS) [8]. The concept underlying this “syndrome” is that certain subjects with obesity are somehow protected from the MS consequences. If true, this would justify a more lenient medical approach towards the metabolically healthy obese patient, particularly considering the current limited success in attaining a sustained reversal or even amelioration of obesity. Although a subset of subjects with obesity do not show the full breadth of traditional risk factors associated with insulin resistance and/or disturbed blood pressure and lipid profile, the classification of NMO eventually evolved so as to allow the inclusion of subjects showing one or two components of MS [9,10]. Even at face value, many such subjects missing just one feature to be labeled as MS patients can be hardly viewed as having normal metabolism [11]. Still, the entity of a metabolically healthy phenotype has gained growing popularity and even received some mechanistic support by observations that it is apparently linked to high adiponectin concentrations relative to the metabolically impaired obese [12,13,14]. It should be mentioned that there are additional criteria to define NMO that are based on tracking the lack of hospitalization for somatic disorders until mid-life in spite of obesity [15], and this definition is appropriate for studies that include follow-up (prospective studied). Although NMO has been claimed to be fairly common, its reported prevalence varies considerably, from just 3.3% all the way up to 32.1% in men [14] and ranging from 11.4% to 43.3% in women [12]. Given the diversity of the factors involved in the evolution of obesity-related disease, inter-subject variability in susceptibility to and/or protective mechanisms from the ill effects of excessive fat tissue appear plausible. An alternative perception of NMO, however, is that the presence of normal metabolism in obesity is temporary and anomalies emerge over time, as seen in the Nurses' Health Study [16]. The latter study showed that 84% of metabolically healthy obese women converted to unhealthy phenotypes compared to 68% of women with normal weight, after 20 years.

In our scenario, then, the presence of just 1–2 MS components may not reflect good metabolic health but rather heralds the subsequent appearance of additional anomalies as a function of time, increasing body mass index (BMI) over time, and/or age. Apparently metabolically healthy subjects with obesity may be in transition to a clearly unhealthy phenotype following longer “incubation time”, further fat mass expansion [17], and/or aging. Recent evidence that indicates that even presumably healthy obesity is linked to increased long-term morbidity and mortality is in accord with this concept [18,19]. 

In the present report, we examine metabolic health across a wide range of ages in a workplace-based health screening cohort. Rather than allowing the slippage of 1–2 “dysmetabolic” features into the definition of NMO, we set out to fully characterize the metabolic state of a large cohort of medically classified normal subjects with a full weight range ranging from clearly lean subjects all the way to grade III obesity (BMI 18.5–46 kg/m^2^). Subjects treated for any pre-identified disease (e.g., diabetes, hypertension) were a priori excluded. We aimed to scrutinize the (a) relationships between BMI and the number of MS components to their numerical continuous values from a very low BMI and upwards; (b) the prevalence of healthy obesity (no MS component, MS = 0) among subjects with obesity and untreated subjects; (c) the effect of gender on the prevalence and distribution of MS components; and (d) the prevalence of hypertension in women and men as a function of the number of MS components.

## 2. Materials and Methods

### 2.1. Study Population

A total of 19,328 persons attending the Tel Aviv Sourasky Medical Center for a routine health examination between September 2002 and March 2013 as part of periodic health check-ups were asked to participate in the Tel-Aviv Medical Center Inflammation Survey (TAMCIS) (for details regarding this cohort, see also [20]). Participants included mostly subjects from large workplaces in the Tel Aviv area in Israel as well as private subjects interested in undergoing a health screening program. They were recruited individually by an interviewer while they waited for their clinical examination. Each participant signed a written informed consent form, which was approved by the institutional review board of Tel Aviv-Sourasky medical center. To qualify for inclusion in this retrospective analysis, subjects had to (1) agree to participate in this study and sign an informed consent form, and (2) for the MS = 0 (no MS component), we included only subjects who had no history or documented diagnosis of or who had not received medical treatment for hypertension, dyslipidemia, and diabetes, leaving a total of 14,093 subjects for analysis. Subjects already treated medically for any of their MS components were not included in the MS = 0 group. Our aim was to study subjects who were free of any MS component rather than subjects that are well treated and thus well controlled. The rest of the subjects were included in the analyses and were assigned to MS1-5 according to their health status.

### 2.2. Study Outcomes

Overnight fasting blood samples were collected from all respondents, followed by a baseline medical history and physical examination, including detailed information on drug therapy and measurement of BMI and WC. Blood pressure was measured by a nurse three times on the left arm after at least 10 min of rest in the seated position, using a clinically validated automated oscillometric sphygmomanometer (Welch Allyn, vital signs monitor 300 series) with an appropriately sized cuff. The average of the last two readings was used for analyses.

Body mass was obtained using an electronic scale with a 200 kg capacity and accuracy to 5 g. Stature was measured using a 2 m stadiometer coupled to the scale (Health O Meter). BMI was calculated according to guidelines of the World Health Organization (WHO) [21]. WC was measured using an inelastic metric tape at the midpoint between the last palpable rib and the iliac crest. To define metabolic normalcy as assessed by WC, measurements were analyzed twice, once in accord with the ATP III, US-based criteria for normalcy (<88 cm for women; <102 cm for men), and additionally based on the IDF-proposed lower cut-off criteria for normalcy in subjects with “Europid” ethnicity [21,22](<80 cm for women; <94 cm for men).

Blood samples were collected after a 12 h fast in vacuum blood collection tubes with gel and centrifuged for 15 min at 4000× *g* to obtain serum. Fasting glucose was determined with the glucose oxidase method by using an autoanalyzer (Beckman Instruments, Fullerton, CA, USA). Total serum cholesterol was measured with the Roche/Hitachi 747 Analyzer (Roche Diagnostics, Mannheim, Germany) and the Raichem Kit (Reagents Applications, San Diego, CA, USA). Glycohemoglobin (HbA1c) was measured using reagents, calibrators, and control materials from Bayer Diagnostics (Berks, England) on an ADVIA 1650 chemistry analyzer according to the manufacturer's instructions. HbA1c was reported as the ratio between its concentration and that of total hemoglobin (%HbA1c).

### 2.3. Statistical Analysis

We conducted statistical analysis using SPSS (Version 21.0; IBM, Armonk, NY, USA) and STATISTICA (version 8.0, TIBCO Software Inc., Palo Alto, CA, USA) software. Descriptive statistics were used to summarize the data. Continuous variables were expressed as mean ± S.D., while qualitative variables were presented as the number of participants and percentage. We used bars and line-plots with or without stratification to selected variables and with regression lines to compare two continuous variables. We used chi-square to compare the gender effect on the order of detection of components of the MS. To characterize the study population with a BMI lower or higher than 30 kg/m^2^, we used factorial analysis of variance with two factors, gender and BMI, without repeated measures. This allowed us to compare the different parameters between men and women who were NMO or NO-NM. We followed up the analyses with the “HSD for unequal N” post hoc test, with an alpha level set to 0.05 for all tests.

## 3. Results

### 3.1. Prevalence of Completely Metabolically Healthy Subjects as a Function of Gender and BMI Groups 

As shown in Table 1, from a total of 19,328 originally screened, 6391 subjects according to the ATP III criteria and 5174 subjects according to the IDF criteria were metabolically truly healthy (absence of any component of the MS; MS = 0 with no drug treatment).

Of this “completely metabolically healthy” cohort, which included 3903 men and 2167 women according to the ATP criteria, 122 men (3.1%) and 51 women (2.4%) were obese. According to the IDF criteria, 3074 men and 2130 women were “completely metabolically healthy”, of whom 23 men (0.8%) and 7 women (0.3%) were obese.

In all, 55% of the non-obese population (BMI < 30 kg/m^2^) were MS = 0 according to the ATP and 43% according to the IDF criteria. Among the obese population (BMI ≥ 30 kg/m^2^), only 7.5% were MS = 0 according to the ATP and 1.4% according to the IDF criteria. Thus, even with the a priori exclusion of subjects with defined metabolic disease, the lack of any components of MS is uncommon in the obese. Furthermore, only half of the non-obese subjects were true MS = 0.

### 3.2. Normometabolic Non-Obese (NO-NM) Subjects Have a More Favorable Cardiometabolic Profile Than Normometabolic-Obese (O-NM) Subjects

As shown in Table 1, for all the classical modifiable cardiovascular risk factors that were recorded in this cohort, mean levels were more favorable in the NO-NM than in the O-NM subgroups: particularly noteworthy were lower systolic blood pressure (3/6 mmHg (ATPIII/IDF)); lower fasting glucose, triglycerides, and LDLc; and higher HDLc. As rough surrogate markers for the degree of liver steatosis, all liver enzymes were lower in the non-obese (NO-NM) than in the O-NM (obese normometabolic) obese healthy group, as was also hsCRP (*p* < 0.05–0.01). Overall, then, even truly O-NM have a less favorable cardiometabolic risk profile than NO-NM, though their actual levels of glucose and lipids are within the normal range (Table 1, US-ATPIII and IDF standards).

### 3.3. BMI Is Continuously Related to Metabolic Measures in Normometabolic Subjects

**Figure 1:**
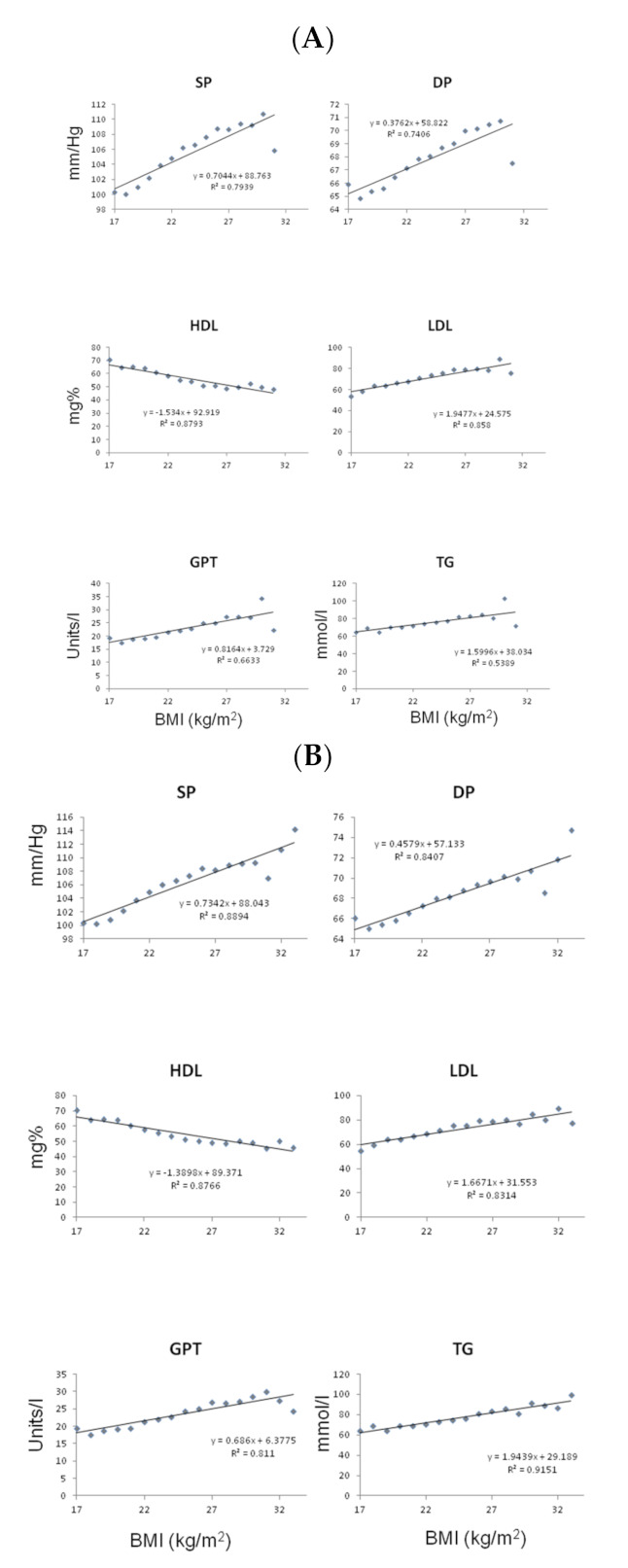
Representation of the numerical values of different components of the metabolic syndrome or associated parameters and BMI in subjects free of any of the components of the metabolic syndrome (MS = 0): (**A**) according to the IDF criteria and (**B**) according to the ATPIII criteria. Abbreviations: DP—Diastolic pressure (mmHg), GPT—Glutamate pyruvate transaminase (U/L), HDL—High-Density Lipoprotein, LDL—Low-Density Lipoprotein (mmol/L), SP—Systolic Pressure (mmHg), TG—Triglycerides.

As shown in Figure 1, significant linear positive correlations existed between BMI (19–32 kg/m^2^) and blood pressure, glucose, lipid profile, and liver enzymes for subjects with an MS score = 0, after adjustment for age (n = 6548). There was a parallel negative linear correlation between HDLc and BMI.

### 3.4. Young Women with and without Obesity Are Metabolically Protected Relative to Men

A gender-related better overall profile in females relative to males was clearly visible in the O-NM. Higher levels in men were recorded for mean systolic and diastolic pressure (SP, DP; 118 ± 8 vs. 112 ± 9 mmHg *p* < 0.01; 76 ± 6 vs. 72 ± 6 mmHg (*p* < 0.01); triglycerides (1.1 ± 0.31 vs. 0.93 ± 0.3 mmol/L, *p* < 0.05), LDL (3.28 ± 0.69 vs. 2.92 ± 0.64 mmol/L; *p* < 0.01); and lower levels of HDL (1.32 ± 0.2 vs. 1.63 ± 0.28 mmol/L, *p* < 0.01). In parallel to the findings in the NO-NM group, liver enzymes were also higher in men (GPT: 30 ± 18 vs. 18 ± 5 U/L; *p* < 0.01, GOT: 27 ± 19 vs. 20 ± 3 U/L, *p* < 0.01). There was no significant difference, however, in fasting glucose (4.88 ± 0.44 vs. 4.77 ± 0.38 mmol/L, *p* = 0.14), and hsCRP was lower in men (31.42 ± 35.23 vs. 53.35 ± 59.04 nmol/L, *p* < 0.05).

**Figure 2:**
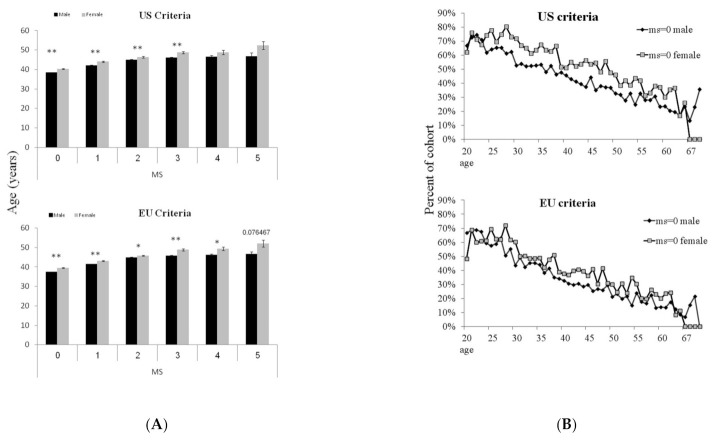

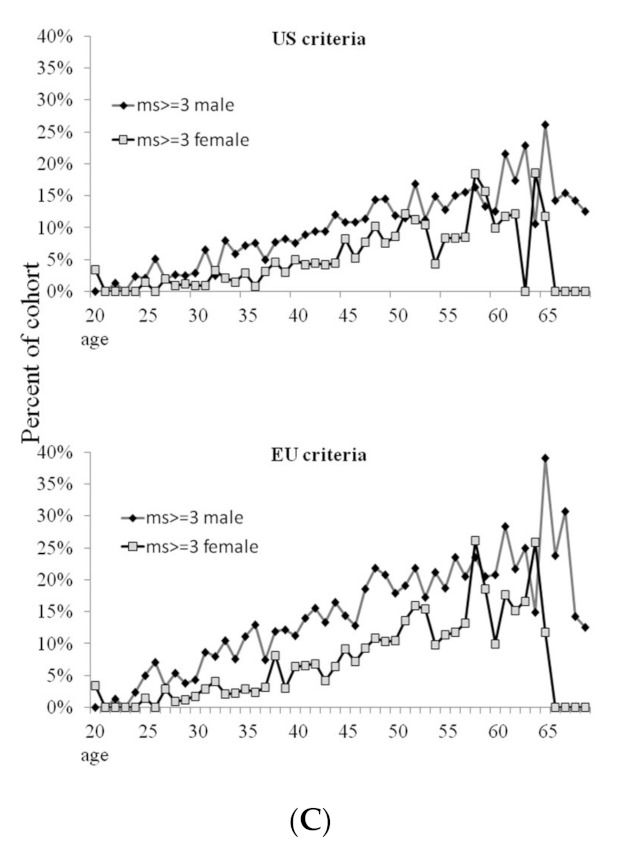
(**A**)**:** Gender and age differences in subjects with and without metabolic syndrome. Average age of men (black bars) and Women (gray bars) for each MS category. (**B**): Gender difference in normometabolic subjects (MS = 0) at all ages (n = 6548, 20–70 years). Average age of men (black diamonds) and Women (gray squares). (**C**): Gender difference in subjects with metabolic syndrome (MS ≥ 3) at all ages. Average age of men (black diamonds) and Women (gray squares). One asterisk describes (*) *p* < 0.05, two asterisk describe (**) *p* < 0.01.

Per each of the six MS categories (from MS = 0 to MS = 5), there was a significant age difference (*p* < 0.05), where men were 2–5 years younger than women (see Figure 2A). Moreover, between the ages of 30 and 50, the fraction of women who did not show any of the MS components was on average ~11% higher compared to men. There was also a ~5 years female to male gap: for example, 52% of men at the age of 37 and 51% of women at the age of 42 years (5 years older) were classified as MS = 0. At the age of 55, this gender difference disappeared (Figure 2B, upper panel). The presence of MS ≥ 3 was also gender/age-related: for example, 8% of men at the age of 40 and 8% of women at the age of 47 years (7 years older) were MS ≥ 3. At the age of 58, this gender gap disappeared (Figure 2C). Although complete “metabolic health” (MS = 0) declines with age in subjects with central obesity, a parallel decrease is clearly discernible even in men and women with normal WC (Figure S1, Supplementary Materials).

**Figure 3:**
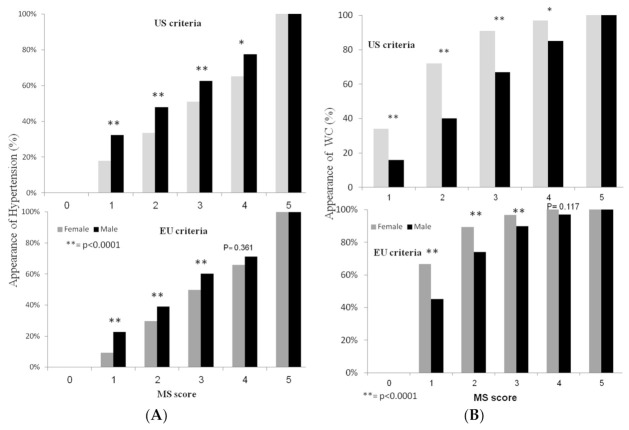
(**A**): *Appearance of Hypertension with the metabolic syndrome components.* The appearance of hypertension according to the MS categories in men (black bars) and women (gray bars). Chi-square test was performed to compare the presence of hypertension between men and women within each MS category; ** represents *p* values < 0.0001 and * represents *p* values < 0.05 (**B**) Appearance of central obesity with the metabolic syndrome components. The appearance of central obesity, evaluated as high waist circumference (WC), according to the MS categories in men (black bars) and women (gray bars). Chi-square test was performed to compare the presence of WC between men and women within each MS category; ** represents *p* values < 0.0001 and * represents *p* values < 0.05.

Another important gender effect was that the order of detection of components of the MS as their number increases is clearly sexually dimorphic: in men, hypertension was the leading, most prevalent component when the MS component number was low, being present in 32%, 48%, 62%, and 74% of men with MS = 1 (χ2 = 100.2, *p* < 0.0001), MS = 2 (χ2 = 38.3, *p* < 0.0001), MS = 3 (χ2 = 9.1, *p* < 0.0001), MS = 4 (χ2 = 3.8, *p* = 0.05), respectively. In women, in contrast, central obesity shows the “earliest” and highest prevalence, as it was present in 32%, 74%, 91%, and 97% of women with MS = 1 (χ2 = 185.7, *p* < 0.0001), MS = 2 (χ2 = 203.6, *p* < 0.0001), MS = 3 (χ2 = 49.8, *p* < 0.0001), MS = 4 (χ2 = 6.6, *p* = 0.01), respectively (Figure 3A,B).

### 3.5. Metabolic Syndrome Components Are Prevalent in Normal BMI and Accumulate as a Function of Increasing BMI and Age

**Figure 4:**
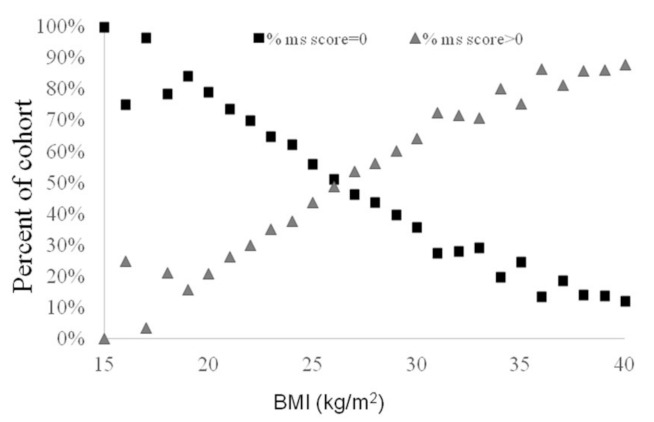
Distribution of normometabolic subjects by BMI. Figure shows the percent examinees with MS = 0 (black diamond) and MS > 0 (gray triangle) as a function of BMI. As BMI increases, the rate of normometabolism decreases. Even with a normal BMI (of 19), 16% of the cohort had at least one component of the metabolic syndrome.

As shown in Figure 4, the association between BMI and MS is such that at a BMI of 19 kg/m^2^, 16% of the cohort had at least one component of the MS, thereby rising linearly with BMI to a point that at a BMI of 29 kg/m^2^ (still within the non-obese range), most (75%) of the participants already had at least one of the MS components. When only subjects with obesity were analyzed (n = 2087; BMI 30 kg/m^2^ or higher), a normometabolic state (MS = 0) was BMI-related and declined from 10% at a BMI of 30 to <1% at a BMI ≥ 36 kg/m^2^.

## 4. Discussion

We report a very low prevalence of “healthy obesity” in a cross-sectional, retrospective study of a large cohort of apparently healthy employees of large workplaces in the metropolitan Tel Aviv area. Since subjects with any medically pre-treated components of metabolic syndrome (e.g., hypertension, diabetes, and hypertriglyceridemia) were a priori excluded from the analysis, the rate of normalcy in subjects with obesity would have been even lower.

The reported prevalence of metabolically healthy obese subjects likely depends on a plethora of factors such as culture-related lifestyle, dietary preferences, and ethnicity-dependent genetic variation in metabolic sensitivity to rising weight as well as the regional distribution of accumulated fat. For example, in one multinational study from 29 countries, East Asians presented the largest accumulation of visceral adipose tissue but the lowest accumulation of deep subcutaneous adipose tissue with increasing adiposity, leading to a particularly high cardiometabolic risk [23]. Similarly, compared with other ethnic groups, South Asians tend to have high fat mass and low lean mass, which likely account for greater levels of insulin and insulin resistance in this group [24]. At times, the impact of ethnic determinants may be counterintuitive and generate confounding effects: in one US study, African-American women demonstrated three-fold odds of prevalent hypertension compared to Hispanic-American women, but among men, BMI, and not visceral adipose tissue, was linked to the variation in hypertension [25]. In this context, the current Israeli population is a multiethnic society of mostly first to seventh generations of immigrants comprising a variety of Ashkenazi and Sephardic Jews with increasing rates of intermarriages and non-Jews, particularly Arabs and Christians of variable ethnic backgrounds.

Another easily discernible factor that affects the perceived rate of metabolically healthy obese subjects is the set of criteria applied to define health: when strict criteria are applied, a fairly low rate of NM obesity tends to be found (e.g., ~7%), whereas rates are fairly high (e.g., 36.6%) if a more permissive approach is used, allowing the inclusion of subjects with only some of the components of MS [9,10].

With a strict approach to the definition of a normal metabolic state, allowing none of the components of MS in our analysis, five major findings emerged. First, “true” metabolic health (“normometabolism”; “MS = 0”) was very rare even among apparently healthy obese (BMI ≥ 30) subjects, reaching ~1% at a BMI ≥ 36 kg/m^2^ and only 7.5% for those with a BMI of 30 kg/m^2^ and higher. Second, even the leanest participants included a significant fraction of subjects with some metabolic impairment: at the low BMI aggregate of 19 kg/m^2^, for example, subjects with MS > 0 comprised ~16% of the cohort, and this rate increased linearly and steeply as a function of BMI throughout the entire range of normal BMI, continuing at a steady increase per BMI unit all the way through the overweight (25–29 kg/m^2^) into the obese range, plateauing from a BMI of 31 kg/m^2^ (Figure 3A). For example, at a BMI of 25 kg/m^2^, ~3% of the screened subjects had MS ≥ 3, and would thus be defined as having MS, which further rises to some 19% in subjects whose BMI is 30 kg/m^2^. Since we a priori excluded subjects receiving any medications, the fraction of subjects with MS in the entire screened population would be necessarily larger. The third key finding is that the rate of MS increases with age, even in lean subjects. Although this is a cross-sectional, not a longitudinal, study, it is still impressive that only a minority of subjects above the age of 55 enjoy complete metabolic health (MS = 0 in 16% of men and 21% of women). Longitudinal studies show that even stable NMO are prone to higher risk for diabetes, cardiovascular disease, and heart failure [26,27]. 

The fourth finding is that women are metabolically protected relative to men, even though excess body fat is apparent earlier in women compared to men. At an older age, however, this gender difference disappears. 

Our data regarding the prevalence of MS and gender are in some disagreement with “The Atherosclerosis Risk in Communities Study” [28], in which female gender was associated with the incidence of metabolic syndrome, MS, among individuals with normal weight, along with other factors such as older age, heavy alcohol consumption, and sedentary behavior. 

The fifth key finding, which is also related to gender, is that hypertension is more prevalent in men with MS1-4 than in women. This may imply that hypertension either appears prior to central obesity in men or that, keeping in mind the android body build, the lesser accumulation of visceral fat, below the threshold definition of increased WC, may already be sufficient to facilitate the evolution of hypertension in men. 

To the best of our knowledge, this is the first study to describe a linear relationship between BMI, ranging from the low normal range to the overweight and obese range (19–39 kg/m^2^), and all the components of the MS. As the number of MS-related anomalies increased with BMI, there were inter-individual differences in terms of which anomaly was the most sensitive to the rise in BMI. For some individuals, a rise in blood pressure may be the “first” to emerge, while for others, it may be fasting glucose or triglycerides. Furthermore, the order in which these anomalies emerge is not random. Hypertension is the most prevalent component in MS1-4 in men, whereas increased waist circumference is the most prevalent component in women at the same MS level. This is consistent with studies that show that women have twice the risk of being overweight or obese, which may be attributed to gender-related differences in the ability to compensate for the burden of obesity, such as the phases of the menstrual cycle, hormonal contraception usage, menopause affecting energy expenditure, and substrate utilization [29]. These individual responses may partly explain why there is no specific BMI level above which these parameters start to rise on average. It should be noted that the 3/6 mmHg difference (ATP/IDF) in SBP between non-obese and obese subjects is clinically relevant, as even a reduction of 3 mmHg has a significant impact on cardiovascular outcomes [30,31]. Our results challenge the concept that NMO is a common condition in that they show a linear and age-related rise in the numerical means of all components of MS with increasing BMI across all non-obese BMI categories and an exceptionally low rate of ≤1% of true NMO in subjects with a BMI ≥ 36 kg/m^2^. We would argue that the concept of metabolically healthy obesity is not merely a misnomer but one with a dual negative health sequel. First, we suggest it provides false assurance to subjects with obesity in whom MS components will appear with the passage of time: according to the ATP criteria, at the age of 30, 20% of obese subjects are metabolically healthy, but at the age of 50, only 5% are metabolically healthy obese; according to the IDF criteria, at the age of 30, only 4% of obese subjects are metabolically healthy, whereas at the age of 50, this rate drops to 1%.

There are several limitations to our report. Firstly, it is based on cross-sectional assessment, which does not allow insight into the passage of time in the studied cohort. This weakness is somewhat compensated for by the relation between MS and age, suggesting that the rate of NMO declines precipitously as a function of age. Secondly, a small percentage of subjects with obesity are normometabolic (MS = 0). Although this is a statistical limitation, it accords with our hypothesis about the paucity of a true normometabolic state within the obese population. The third limitation is that our cohort was constructed from a health screening program and is not a population-based sample. Since the average age of this cohort is ~40, our data can be compared to a published national health survey from Israel showing some gross similarities to our cohort. At the age range of 35–44 years, the mean BMI for women and men were 26.8 and 26.6 kg/m^2^, respectively [32], which are rather close to the overall mean BMI of 25.66 ± 4.01 kg/m^2^ (24.71 ± 4.52 and 26.24 ± 3.55 kg/m^2^ in women and men, respectively) in our study. Additionally, reminiscent of our own cohort, hypertension was more common in men, whereas the rate of increased waist/hip circumference was higher in women. Finally, as we aimed to identify true metabolic health in apparently healthy subjects, we excluded all subjects with medically treated components of metabolic syndrome a priori. Thus, our analysis (by design) is not representative of the general population.

If obese subjects with treated hypertension or diabetes had been included, the rate of healthy obesity would have been even rarer. In summary, our results show not only that NMO is rare in subjects with obesity but also that the risk factors and/or risk markers of MS increase with rising BMI, age, and any other risk factor even within the normal range. The quantitative increase in risk is likely related to future health outcomes. This does not, however, mean that we suggest redefining MS. Rather, we are pointing out that physicians should exercise caution when an MS criterion is added on top of an existing one or when there is an increment in any risk factor even within the normal range.

Finally, there is a gender difference in the distribution of MS components such that from MS1 through MS3, the latter already defining an existing MS, the rate of hypertension is higher in men, whereas the rate of increased WC is higher in women. This difference awaits further study in populations of both similar and different ethnic make-up relative to our cohort.

## 5. Conclusions

Whereas MS is defined by a rigid set of criteria, normal metabolism is harder to define. With increasing BMI, starting at the lower end of the normal range and onward, the parameters comprising the criteria for MS rise continuously and linearly with height-adjusted body weight in a gender-related manner. This cross-sectional study suggests that in men, an early rise in blood pressure and actual hypertension precede the subsequent emergence of additional components of MS. The continuous weight-related change in MS components may argue against the dichotomization of obesity into normal/abnormal in terms of its metabolic and vascular sequels. Furthermore, because the harms of obesity may be delayed but significant, a permissive approach towards the metabolically healthy state seems ill-advised.

## Figures and Tables

**Table 1:** Characterization of the study population according to a BMI lower than 30 (non-obese) and higher than 30 (subjects with obesity). All variables are age-adjusted and presented as mean ± STD. Significance in factorial analysis of variance is depicted for the respective groups as indicated at the bottom of the table.

Normometabolic Obesity BMI ≥ 30 kg/m^2^			Normometabolic Non-Obese Population BMI < 30 kg/m^2^			
Female	Male	All	Female	Male	All	Average ± SD
7	23	30	2123	3051	5174	N IDF
36	121	157	2632	3759	6391	N ATP III
39 ± 10	40 ± 10	40 ± 10	39 ± 10 *	37 ± 10	38 ± 10	Age (years) IDF
43 ± 8 *	40 ± 9	41 ± 9	40 ± 10 *^,$^	38 ± 10 ^$^	39 ± 10 ^$^	Age (years) ATP III
80 ± 7 *	91 ± 6	89 ± 8	58 ± 7 *	75 ± 8	68 ± 11 ^$^	Weight (kg) IDF
77 ± 7 *	92 ± 7	89 ± 9	60 ± 7 *	76 ± 8	70 ± 11	Weight (kg) ATP III
31 ± 2	31 ± 2	31 ± 2	22 ± 2 *^,$^	24 ± 2 ^$^	23 ± 2 ^$^	BMI (kg/m^2^) IDF
31 ± 1	31 ± 1	31 ± 1	23 ± 2 *^$^	25 ± 2 ^$^	24 ± 2 ^$^	BMI (kg/m^2^) ATP III
74 ± 2 *	91 ± 4	89 ± 8	72 ± 5 *	86 ± 6 ^$^	80 ± 9	Waist Circumference (cm) IDF
83 ± 4 *	98 ± 3	94 ± 7	75 ± 6 *^,$^	88 ± 7 ^$^	93 ± 9	Waist Circumference (cm) ATP III
115 ± 15	120 ± 6	119 ± 9	108 ± 10 *	115 ± 9	113 ± 10 ^$^	SBP IDF
113 ± 10	118 ± 9	116 ± 9	108 ± 10 *	116 ± 9 ^$^	113 ± 10 ^$^	SBP ATP III
73 ± 9	77 ± 5	76 ± 6	70 ± 6 *	73 ± 6	72 ± 6	DBP IDF
73 ± 6	76 ± 6	75 ± 6	70 ± 6 *	74 ± 6 ^$^	72 ± 6 ^$^	DBP ATP III
4.9 ± 0.5 *	4.5 ± 0.5	4.6 ± 0.6	4.5 ± 0.5 *	4.7 ± 0.5 ^$^	4.9 ± 0.5	Glucose (mmol/L) IDF
4.8 ± 0.4	4.9 ± 0.4	4.9 ± 0.4	4.6 ± 0 *^,$^	4.8 ± 0.3	4.7 ± 0 ^$^	Glucose (mmol/L) ATP III
0.8 ± 0.3	0.7 ± 0.2	0.7 ± 0.2	0.8 ± 0.3 *	0.9 ± 0.3 ^$^	0.9 ± 0.3 ^$^	Triglycerides (mmol/L) IDF
0.9 ± 0.3 *	1.1 ± 0.3	1.0 ± 0.3	0.8 ± 0 *^,$^	0.9 ± 0 ^$^	0.9 ± 0	Triglycerides (mmol/L) ATP III
2 ± 0.7	2.4 ± 0.6	2.3 ± 0.6	1.8 ± 0.4 *	1.4 ± 0.5	1.6 ± 0.5	HDL (mmol/L IDF
1.6 ± 0.3 *	1.3 ± 0.2	1.4 ± 0.3	1.8 ± 0.3 *	1.4 ± 0.1	1.6 ± 0.1	HDL (mmol/L) ATP III
1.7 ± 0.5	2.3 ± 0.6	2.2 ± 0.6	1.8 ± 0.7 *	2 ± 0.7 ^$^	1.9 ± 0.7	LDL (mmol/L) IDF
2.9 ± 0.6 *	3.3 ± 0.7	3.2 ± 0.1	2.8 ± 0 *	2.9 ± 0.1 ^$^	2.9 ± 0.1 ^$^	LDL (mmol/L) ATP III
43 ± 40	36 ± 41	38 ± 40	28 ± 24 *	34 ± 27	32 ± 26	GOT IDF
20 ± 3 *	27 ± 19	25 ± 17	21 ± 7 *^,$^	24 ± 9 ^$^	23 ± 9 ^$^	GOT ATP III
44 ± 40	35 ± 29	37 ± 32	26 ± 25 *	35 ± 27	31 ± 26	GPT IDF
18 ± 5 *	30 ± 18	28 ± 17	18 ± 8 *^$^	25 ± 12 ^$^	22 ± 11 ^$^	GPT ATP III
6.0 ± 5	3 ± 3	4 ± 4	2 ± 3 *	2.0 ± 3	2 ± 3 ^$^	hsCRP IDF
6 ± 6 *	3 ± 4	4 ± 4	2 ± 3 *	2 ± 3	2 ± 3 ^$^	hsCRP ATP III

Abbreviations: DBP—Diastolic Blood pressure (mmHg), GOT—Glutamate oxaloacetate transaminase (U/l), GPT—Glutamate pyruvate transaminase (U/L), HDL—High-Density Lipoprotein, hsCRP—High-sensitive C-Reactive Protein (mg/L), LDL—Low-Density Lipoprotein (mmol/L), SBP—Systolic Blood Pressure (mmHg), IDF—International Diabetes Federation, ATP III—Adult Treatment Panel. * Indicates *p* < 0.05 between males and females within the Normometabolic non-obese population BMI < 30 kg/m^2^ and between males and females within Normometabolic non-obese population BMI < 30 kg/m^2^. ^$^ indicates *p* < 0.05 significant difference between the Normometabolic non-obese population BMI < 30 kg/m^2^ and Normometabolic obese population BMI > 30 kg/m^2^ in respective groups (all, males, and females). First black line per each parameter presents data according to the IDF; Second gray line presents data according to ATP III.

## Data Availability

Data will be provided upon request.

## Supplementary Materials

The following are available online at https://www.mdpi.com/article/10.3390/biology12050719/s1, Figure S1: The decline of metabolically healthy obese subjects (men and women) with age.
