# Supplementary figures and images for: Metabolically Healthy Obesity Is a Misnomer: Components of the Metabolic Syndrome Linearly Increase with BMI as a Function of Age and Gender
Source: Biology (Basel). 2023 May 15;12(5):719. doi: 10.3390/biology12050719 (PMC10215588; doi:10.3390/biology12050719)

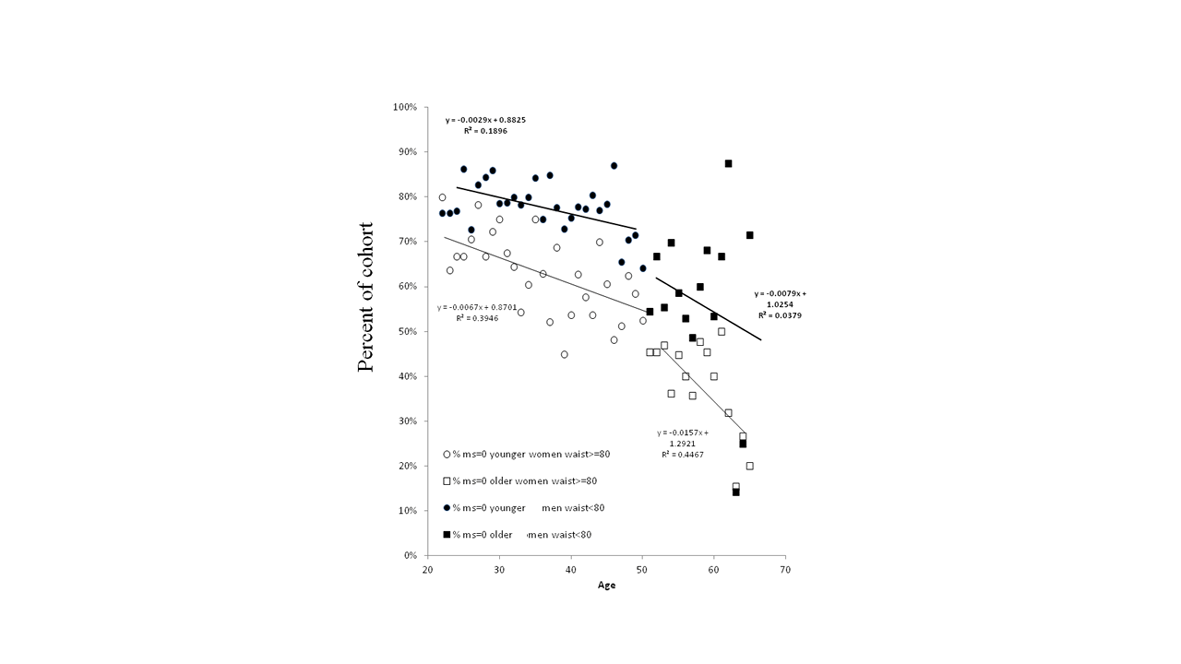

Supplement: Supplementary file 1 [file biology-12-00719-s001.zip › biology-2398960-supplementary.tif]
